# Rapid assessment of myocardial scar and viability in ischemic heart disease: a cardiovascular magnetic resonance study

**DOI:** 10.1186/1532-429X-11-S1-P88

**Published:** 2009-01-28

**Authors:** Didier Locca, Chiara Bucciarelli-Ducci, Jonhatan Lyne, Marinella Chapparo, Giuseppe Ferrante, Rory O'Hanlon, Sanjay Prasad, Peter Gatehouse, Peter Drivas, Rick Wage, David Firmin, Dudley Pennell

**Affiliations:** Royal Brompton Hospital, London, UK

**Keywords:** Cardiovascular Magnetic Resonance, Late Gadolinium Enhancement, Single Shot, Myocardial Scar, Late Enhancement

## Introduction

One important use of Cardiovascular Magnetic Resonance (CMR) is to visualize the extent of myocardial infarction. Inversion recovery preparation with segmented gradient echo readout (IR-GRE) is considered the gold standard sequence for the detection of late gadolinium enhancement to visualise myocardial infarction. However this technique requires multiple breath-holds and is time consuming. A reliable single shot readout multislice inversion recovery imaging sequence would permit rapid viability imaging improving scanning efficiency.

## Purpose

The aim of this study was to compare the diagnostic accuracy of the multislice single shot SSFP inversion recovery sequence (IR-SSFP) against multiple breath-hold IR-GRE images in detecting the presence or absence of late enhancement.

## Methods

We studied 86 consecutive patients undergoing a CMR viability assessment. Images were acquired 10 minutes following intravenous injection of 0.1 mmol/Kg gadolinium-DTPA.

## Results

Images were analyzed by two blinded observers for the presence or absence of late myocardial enhancement. Both techniques identified the presence of myocardial infarction in 21 patients. Interobserver variability was -1.370 (CI -11.327 to 8.588) for GRE and -1.707 (CI -9.849 to 6.435) for SSFP. The coefficient of repeatability was 42.54 fro GRE and 41.94 for SSFP. The interobserver agreement in the estimate of viability was 0.84 (0.11) for GRE and 0.95 (0.11) for SSPF technique.

## Conclusion

Despite its lower spatial resolution, the IR-SSFP single shot multislice imaging sequence was equally capable of detecting the presence or absence of late enhancement compared to the single slice GRE sequence. Clinically, the IR-SSFP sequence can be used to perform a rapid assessment of the presence or absence of late myocardial enhancement to determine myocardial scar and viability.

